# Little Journey: a phase III randomised controlled trial of a psychological preparation and education smartphone application for management of paediatric perioperative anxiety compared with standard care in children undergoing ambulatory surgery – study protocol

**DOI:** 10.1136/bmjopen-2024-090696

**Published:** 2025-02-26

**Authors:** Christopher Evans, Georgia Bercades, Gareth Ambler, Matthew Wilson, Chris Brew-Graves, Cinzia Baldini, Nazma Begum-Ali, Norman R Williams, Mark Emberton, Matthew Fenton, Daisy Fancourt, Mohini Samani, Monty Mythen, Suneetha Ramani Moonesinghe

**Affiliations:** 1 Centre for Perioperative Medicine, Research Department of Targeted Intervention, University College London, London, UK; 2 Critical and Perioperative Care Theme, NIHR University College London Hospitals Biomedical Research Centre, London, UK; 3 Applied Statistics, University College London, London, UK; 4 Health Services Research, University of Sheffield, Sheffield, UK; 5 Division of Surgery and Interventional Science, University College London, London, UK; 6 University College London, London, UK; 7 Surgical Interventional Group, University College London, London, UK; 8 Department of Urology, University College London Hospital, London, UK; 9 Great Ormond Street Hospital for Children, London, UK; 10 Department of Behavioural Science and Health, Department of Epidemiology and Public Health, University College London Research, London, UK; 11 NIHR CRN West Midlands Young Persons Steering Group, Stafford, UK; 12 Anaethesia, University College Hopital, London, UK; 13 National Institute for Health Research's Central London Patient Safety Research Collaboration, University College London Hospitals NHS Foundation Trust, London, UK

**Keywords:** Paediatric anaesthesia, Anxiety disorders, Virtual Reality, Paediatric surgery, Randomized Controlled Trial

## Abstract

**Introduction:**

Children having surgery, and their parents, commonly have anxiety in the preoperative period, and this may impact longer-term health and quality of life. Psychological preparation can be expensive and time-consuming, and the type and effectiveness of preparatory interventions are variable. The aim of this randomised controlled trial (RCT) is to evaluate the clinical effectiveness of a preoperative smartphone psychological preparation application with virtual reality (VR) capability (the ‘Little Journey app’ (LJ)), at reducing anxiety and its sequelae in children and their carers.

**Methods and analysis:**

Multicentre, assessor-blinded, two-armed, parallel group, RCT in children aged between 3 and 12 years, undergoing ambulatory surgery and receiving their first general anaesthetic. Randomisation is one-to-one between an intervention and a control arm. Participants in the intervention arm are provided with access to the LJ app and a low-cost cardboard VR headset (to be used with a smartphone) to use in the weeks leading up to their operation. Children in the control arm receive the same VR headset and suggestions of unrelated VR games to play, but no access to the LJ app. To improve accessibility, smart devices are provided to children whose families do not have a smart phone, and the app content has been translated from English into multiple languages. Both groups receive standard perioperative care at the hospital where they are having treatment. The primary outcome measure is the modified Yale Preoperative Anxiety Scale-Short Form applied by independent blinded observers, immediately before induction of general anaesthesia. Secondary outcomes include process measures, psychological and socioeconomic outcomes for both children and parents/carers. The planned sample size was 304 participants, including an anticipated 15% attrition rate. An interim analysis was conducted when the trial was temporarily paused because of the COVID-19 pandemic, at which point 119 participants had been recruited. The trial steering committee and data monitoring committee recommended continuation of the trial, but the sample size was increased to 596 to account for differences between the previously anticipated and actual outcomes of recruited participants.

**Ethics and dissemination:**

The study was approved by Surrey Borders—Research Ethics Committee 251219, and all participating sites were in England. Results will be presented in academic manuscripts and presentations and summarised for diverse audiences (including clinicians and patients/public) in podcasts, infographics and other multimedia formats.

**Trial registration number:**

NCT03797716.

**STRENGTHS AND LIMITATIONS OF THIS STUDY**

- This is a multicentre randomised clinical trial which is evaluating the effect of a simple, scalable intervention at improving patient-centred clinical outcomes in a group of patients who are generally underserved by clinical research.
- Participants are recruited from all surgical specialities with significant day surgery rates, and the exclusion criteria are limited to reduce the risk of extraneous factors (prior experience of general anaesthesia or diagnostic uncertainty) influencing anxiety rates in children.
- Blinded to allocation, the primary outcome is adjudicated by independent observers who have received high-quality training in how to measure anxiety using a previously validated classification of anxiety.
- Owing to the type of intervention delivered, blinding of participants is not possible, but independent observers and data analysts are blinded throughout.

## Introduction

 Anxiety before an operation is common and has an impact. Over one-third of adult patients state that anxiety is the worst thing about having surgery, and it has a greater impact on patient experience than pain, postoperative nausea and vomiting and other symptoms.^1^ Anxiety affects between 50% and 75% of children on the day of surgery.^2^,^4^ Risk factors include the age of the child, personality traits such as low sociability and high impulsivity, parent anxiety, and previous negative healthcare experiences.^5^,^8^ Multiple studies in children have shown links between perioperative anxiety and poor compliance with induction of anaesthesia, emergence delirium and adverse postoperative outcomes such as poor wound healing, regressive behavioural changes and increased analgesic requirements.^29^,^12^

Emotion-focused coping techniques such as the use of distraction interventions (books, toys, clown doctors) and low-sensory environments have been shown to reduce children’s preoperative anxiety.^9 13 14^ Similarly, problem-focused coping techniques, such as preoperative education through hospital tours, information booklets and play therapy, have been shown to be effective when used in isolation or as combined interventions.^515^,^18^ More recently, advances in technology and their availability have meant that the use of smartphones and hand-held tablets is becoming increasingly popular methods of delivering emotion-focused and problem-focused coping interventions.^319^,^22^

The ubiquity of smartphones brings the opportunity to provide interactive and engaging information designed for children and their parents, delivered in their own home. The development of any new preoperative educational intervention should be tailored to the age and level of understanding of the child,^18 23^ delivered at a suitable time to allow for the information to be processed and coping strategies developed, depending on the age of the user,^24^ linked with parental information and provide children with information about each step of the hospital journey.^11^ The Little Journey (LJ) app was developed to support preoperative preparation in children between 3 and 12 years of age, adhering to these design principles.

This manuscript describes the protocol for a randomised controlled trial (RCT) to evaluate the clinical effectiveness of the LJ app at reducing anxiety in children undergoing surgery under general anaesthesia.

## Methods

This study protocol follows the Standard Protocol Items: Recommendations for interventional Trials guidelines.^25^

### Design

The LJ Trial (hereafter known as the LJ Trial) is a multicentre, assessor-blinded, two-armed, parallel group, RCT of a virtual reality (VR) psychological preparation app (LJ app) in children aged 3–12 years old undergoing ambulatory surgery.

### Research questions

The primary aim of the trial is to evaluate the clinical effectiveness of the LJ VR psychological preparation app (known hereafter as the LJ app) in children undergoing ambulatory surgery.

The research questions are as follows:

1. Does provision of access to the LJ app reduce anxiety in children before general anaesthesia for ambulatory surgery?
2. Does provision of access to the LJ app reduce other psychological measures of harm, including post-hospital discharge behavioural changes?
3. What is the acceptability to parents/children of the LJ app?
4. Does the timing and frequency of use of the LJ app affect the clinical effectiveness of the intervention?
5. Is the clinical effectiveness of the app consistent across age groups or does it differ?
6. Are parental anxiety levels affected by having access to the LJ app?
7. Does having access to the LJ app affect use of premedication anxiolysis or sedation in children attending for ambulatory surgery?
8. What relationship is there between preoperative anxiety and use of rescue analgesia and antiemetic medication in the recovery period immediately after ambulatory surgery in children?

### Inclusion criteria

1. Children aged between 3 and 12 years of age on the date of parental consent to participate in the trial.
2. Those undergoing surgery planned to be conducted as a day case (surgery is defined as any therapeutic procedure taking place under the care of an anaesthetist and surgeon or dentist).
3. Requiring general anaesthetic (must be their first general anaesthetic).
4. Both the child and parent are able to speak/understand one of the languages available on the app.
5. American Society of Anesthesiologists’ physical status class I–III
  - Class I: A normal healthy patient.
  - Class II: A patient with mild systemic disease.
  - Class III: A patient with severe systemic disease.

Surgery is defined as any procedure occurring in a theatre under the care of a surgeon or dentist and an anaesthetist.

### Exclusion criteria

1. Children aged less than 3 years of age or more than 12 years’ old on the date of parental consent.
2. Any child and/or parent who refuses to be part of the study.
3. Patients and parents who do not speak one of the languages which are available on the app.
4. Children undergoing diagnostic procedures (eg, scans, cardiac catheterisation).
5. Any child with visual or hearing impairments significant enough to prevent use of the intervention will be decided on case-by-case basis.
6. American Society of Anesthesiologists physical status class IV–VI.

- Class IV: A patient with severe systemic disease that is a constant threat to life.
- Class V: A moribund patient who is not expected to survive without the operation.

To improve accessibility, smart devices are provided to children whose families do not have a smartphone, and the app content has been translated from English into six other languages.

### Screening, recruitment and consent

**Figure 1:**
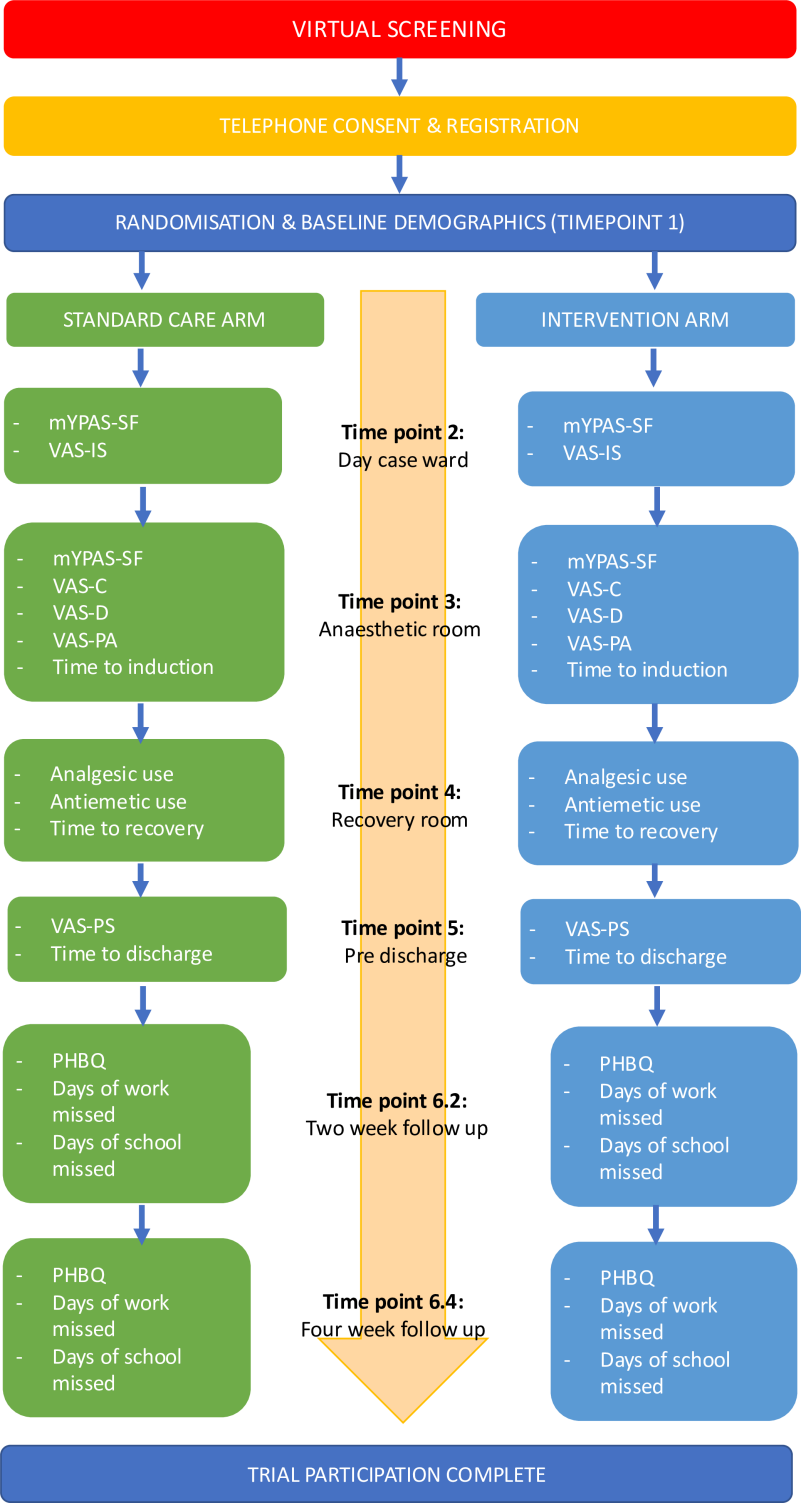
Trial flow diagram. m-YPAS-SF, modified-Yale Preoperative Anxiety Scale-Short Form; PHBQ, Post-Hospital Behavioural Questionnaire; VAS-C, Visual Analogue Scale-compliance; VAS-D, VAS-distress; VAS-PA, VAS-parent anxiety; VAS-PS, VAS-parental satisfaction.

The trial flow is summarised in figure 1 and table 1.

**Table 1:** Study screening, enrolment and randomisation time point

		Study period						
	**Virtual clinic**	**Pre assessment clinic**	**Day of surgery**				**Postdischarge**	
**Timepoint**	**Pre-T1**	**T1**	**T2**	**T3**	**T4**	**T5**	**T6 (2weeks)**	**T6 (4weeks)**
Screening								
Screening	X							
PIS distribution	X							
Enrolment								
Informed consent		X						
Eligibility assessment		X						
Randomisation		X						
Interventions								
Intervention distribution		X						
Little Journey app+VR headset		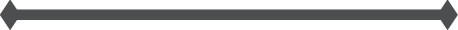						
Standard of care+VR headset		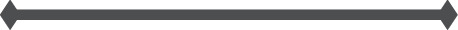						

PISparticipation information sheetVRvirtual reality

Due to the COVID-19 pandemic, preoperative assessment clinic (PAC) appointments for children undergoing ambulatory surgery have, in general, moved from face-to-face appointments to telephone consultations. To enable consent and enrolment of suitable participants undergoing either telephone or face-to-face preoperative assessment, two consent pathways were developed:

### Pathway 1: remote consent

Participants are prescreened virtually through assessment of those placed on a waiting list for day case surgery at participating sites. Prescreening is based on the age of the child, and contingent on there being a minimum of 9 days between telephone consent and the day of planned surgery. This window has been developed to provide a minimum of 5 days of potential use time for the LJ app (allowing 2–3 days for the headset to arrive with the participant).

All eligible participants’ parents/carers are sent a participation information sheet (PIS, see online supplemental material) at least 48 hours before being approached for consent, and a copy of the consent form (see online supplemental material), by email or post. Children are also sent an information leaflet tailored to their age. A minimum of 3 working days will be provided to the parent/carer, between the research team sending them the PIS and subsequently being approached for telephone consent. Telephone consent follows a preapproved script with opportunity provided for the parent/carer to ask questions. Families are provided with a copy of the consent form to refer to during the conversation. Telephone consent is documented on stage one of the consent form and placed in the patient notes. Written confirmation of consent (stage 2 of the consent form) is sought when the patient and carer come to hospital for surgery and before the time of transfer from the ward/outpatient area to the operating department. A copy of the signed consent form is provided to the participant’s parent/carer and in the clinical notes; the original is retained in the investigator site file.

If available, at the time of consent, an assent form will be completed for children aged between 8 and 12 years of age.

### Pathway 2: face-to-face consent

Participants are prescreened at the time of adding to a waiting list for a PAC appointment. Again, prescreening will be based on the age of the child. All eligible participants’ parents/carers are sent a PIS by email or post at least 3 working days before the planned PAC appointment. Children are also sent an information leaflet tailored to their age. Parents/carers may also be telephoned by a member of the research team informing them that the study is taking place, signposting them to the PIS and informing them that a member of the research team will be at the PAC hoping to speak to them to offer the opportunity to take part in the trial.

At the PAC, children and their carers will undergo the standard evaluation and explanation of surgery and anaesthesia by a healthcare professional. This healthcare professional will ask if the parent and child are willing to speak with a member of the research team about the trial. If agreed, a member of the research team will then approach the parents/carers and children with a verbal explanation for the research and give them an opportunity to gain further information. If the parents/carers are content with the information provided, they will be asked to provide written consent for them and their child to be entered in the trial (online supplemental material).

### Intervention

The intervention is a bespoke psychological preparation app (the LJ app) which was developed to prepare children 3–12 years of age for ambulatory surgery. The app was designed by Dr Chris Evans and Sophie Copley and developed by LJ. The LJ app can be downloaded for free from the Apple Store or Google Play store and is designed for use in the 2 weeks approaching surgery. As it is designed for children to use, it requires no training for use and is intuitive in its setup and use with or without a VR headset. The usability of the LJ app was assessed in a single centre study at University College London Hospital, and also tested and deemed easy to use by the West Midlands National Institute for Health Research Young Persons Advisory Group.

The LJ app allows children to explore 360° hospital environments familiarising and desensitising them to the rooms and staff that they will see on the day of surgery. It uses a variety of behaviour change techniques including goals and planning, social support, shaping knowledge and regulation. These are delivered through a variety of features such as the provision of procedural and sensory information, a therapeutic game and child-narrated relaxation exercises—all with the aim of lowering preoperative anxiety and uncertainty.

The LJ app can be used in 2D mode on a hand-held tablet or smartphone or in 3D mode with a smartphone that is inserted into a low-cost flat pack cardboard VR headset. Children are provided with the headsets to use at home.

If randomised to the intervention, the parents/carers are provided with an access code for the LJ trial app. An animation plays within the content of the app showing users how to insert a smartphone into the VR headset. When users first log in, they are asked to enter a hospital access code, which tailors the content specific to their local hospital. Children can then select from two groups of animated hospital staff who they would like to show them around the hospital and from a selection of languages if English is not their first language. They are then taken on a VR tour of their upcoming hospital visit, showing them the different hospital areas that they will visit and introducing them to the staff and pieces of equipment they will meet on the day of surgery. Children can ‘visit’ the day-surgery ward, anaesthetic and recovery rooms of the hospital where their operation will occur while in their own home. As the child explores the three hospital areas, they are introduced to animated characters of staff who explain what will happen in each area, about the equipment that will be used there and how they might feel. Using head tracking technology, the child triggers the animated characters in each area by looking at them; this means they control the pace of their learning and the speed at which they progress through each area. The preparatory tool follows a preset storyline reflecting what will happen on the day of their operation, from admission to discharge.

The LJ app can be used as many times as the child or parent/carer wishes before their operation. To maintain engagement, it has been designed as a game with in-app incentives and rewards for its use, such as the unlocking of new areas and characters. When users first enter the LJ app, they are asked to insert a date of surgery—if known—and the child’s age. This information is used to further tailor the content of the LJ app and triggers a notification system reminding parents/carers when to use the LJ app. If children are 3–6 years old, the LJ app sends an in-phone notification 2 and 4 days before their date of surgery suggesting that this is the optimum time to use the LJ app. For children older than 6 years of age, notifications are sent at 2 weeks and 7 days before the date of surgery. In addition, information is also provided to the parents/caregivers through drip-fed snippet information articles, checklists of what to bring, fasting guidelines and key hospital information such as contact information in case of intercurrent illness.

Other than being provided with access to the app and with a cardboard headset, participants allocated to the intervention arm will receive the same perioperative management as the standard care arm.

### Comparator

Participants assigned to the comparator arm receive standard care throughout their perioperative pathway. The definition of standard care at each trial site will be recorded through a questionnaire at the SIV, prior to commencement of recruitment to the trial. A typical perioperative pathway in most National Health Service hospitals would include meeting a specialist nurse in the PAC (if face-to-face or a phone consultation at least with the parent/carer); a preoperative anaesthetic and surgical consultation on the day of surgery; interaction with play specialists on the day of surgery; and distraction interventions such as hand-held tablets during the induction of anaesthesia. Participants may undergo either an inhalation or intravenous induction depending on the primary management plan and be given premedication at the discretion of the responsible anaesthetist.

Participants in the comparator arm will also receive a flat pack VR cardboard headset and information will be provided about a list of VR games which are freely available from online app stores, which can be played by the child, but which are completely unrelated to surgery or healthcare more generally. The rationale for this was that, during single-centre pilot work, it was found that children randomised to the comparator arm were very disappointed by not being offered ‘the game’. Therefore, to counter this disappointment and minimise, the impact that this might have on the trial outcome, a VR headset is provided.

### Randomisation

All eligible and consented participants will be randomised after baseline assessment to one of two arms. Randomisation is performed using Sealed Envelope, an online software application frequently used in clinical trials. Randomisation will use minimisation to ensure balance across study sites and three groupings of surgical procedures: ear, nose and throat, dental and maxillofacial; urology and gynaecology; and general surgery, orthopaedics and other procedures.

### Blinding

Participants will be unblinded to their allocated trial arm. The member of research staff who performs randomisation will not undertake any further data collection for this patient, as they will not be blinded to the allocation.

Independent observers blinded to the participants allocated trial arm, and who have undertaken the training required to complete the modified-Yale Preoperative Anxiety Scale-Short Form (m-YPAS-SF) assessment will complete data collection on the day of surgery. (see outcome measures section). All efforts will be made to maintain the blinding of the anaesthetic team and independent observer to the allocated study arm, which should be feasible, as the intervention is delivered in the patient’s home.

In the event of accidental unblinding (eg, divulging of allocation by patients in the presence of the researchers undertaking primary outcome assessments), this is recorded by trial sites as a protocol deviation and reviewed monthly by the trial management group (TMG).

The research staff performing data analysis will also be blinded to the trial arms.

### Outcome measures

#### Primary outcome

The primary outcome is preoperative anxiety in the child undergoing surgery, assessed using the m-YPAS-SF, immediately before induction of anaesthesia. The m-YPAS-SF is a valid tool for the accurate measurement of paediatric perioperative anxiety, with good inter-rater and intrarater reliability.^26^ Children are scored in four categories: activity, vocalisations, emotional expressivity and state of apparent arousal, according to predetermined Likert scale observations. Each category score is divided by its highest possible score, before being added together; these scores are then divided by four and multiplied by 100, giving a value between 22.92 and 100. Higher values indicate greater anxiety levels, with scores greater than 30 typically representing clinically significant anxiety and those above 40 representing severe anxiety.^27^

To ensure high inter-rater reliability across all sites and over the whole trial recruitment period, we have created an e-learning module, modified from Jenkins *et al*’s proposed method.^26^ This provides assessor training and enables the testing of inter-rated reliability at multiple time points. For the training, trainee observers watch an introductory lecture outlining the creation of the mYPAS-SF, before observing and scoring 10 real-life case videos. After comparing their scores to five expert scorers, they can listen to explanations as to how and why each expert scored that case. Following this, the trainee observers undertake a test, scoring five new case videos. To pass, they must achieve a Kappa score of 0.7 in each of the four domains to be approved as a data collector.

All sites were required to provide at least two research staff who had passed the training before site opening was permitted; furthermore, after our trial pause because of the COVID-19 pandemic, all trial staff were required to repeat the end-of-training test and re-do the training if required to evidence their competence at undertaking the primary outcome assessment.

#### Secondary outcomes

A combination of parent-reported, independently assessed and objective secondary outcomes are collected on the day of surgery and then at 2 and 4 weeks after surgery (table 2).

**Table 2:** Outcome measurement schedule

	Study period						
	**Preassessment clinic**	**Day of surgery**				**Postdischarge**	
**Timepoint**	**T1**	**T2**	**T3**	**T4**	**T5**	**T6 (2 weeks)**	**T6 (4 weeks)**
Assessments							
Modified Yale Pre-operative anxiety scale- Short-form	**X**	**X**	**X**				
VAS-Parent Anxiety	**X**		**X**				
VAS-satisfaction with informatio		**X**					
VAS-compliance			**X**				
VAS distress			**X**				
Time to induction			**X**				
Analgesic use				**X**			
Antiemetic use				**X**			
Time to recovery				**X**			
VAS-parent satisfaction with care					**X**		
Adverse events questionnaire					**X**		
Time to discharge					**X**		
Unplanned admission					**X**		
Unplanned cancellations					**X**		
Post Hospital Behaviour Questionnaire						**X**	**X**
Number of days of work/school missed						**X**	**X**

VASVisual Analogue Scale

#### Parent reported

Three different 100 mm Visual Analogue Scales (VAS) are used to evaluate the following outcomes:

- Parent satisfaction with information (measured on the morning of surgery after arrival at the hospital).
- Parental anxiety: The VAS-parent anxiety (VAS-PA) is a rapid method of assessing self-reported anxiety levels of parents before surgery. It has been shown to correlate sufficiently with the State Trait Anxiety Inventory (STAI) (r=0.64, p<0.001), the gold-standard research tool used to measure adult state and trait anxiety.^28^ The VAS was chosen as our measure of parental anxiety rather than the STAI - a 40-point questionnaire taking 5–10 min to complete; this is because the STAI was observed in our phase 2 feasibility trial to lead to distress when given to parents/carers immediately following the observation of the induction of anaesthesia in their child. In comparison, the VAS-PA takes 30 s to 1 min to complete and is less likely to lead to distress.
- Parental satisfaction (VAS): found to be more sensitive to differences in levels of parent satisfaction when compared with a 20-point questionnaire used in the feasibility trial. Minimal differences were observed between trial arms, with the majority of participants parents/carers giving full marks in all 20 questions.

Prior to discharge home, parents/carers in both trial arms are also asked to complete a questionnaire assessing if they have used the VR headset and if they or their child developed any adverse symptoms through its use. Similarly, if the child was randomised to the intervention, we ask about any reasons for not using the LJ app.

Telephone consultations at 2 and 4 weeks after surgery ask the following information of parents/carers:

- The Post-Hospital Behavioural Questionnaire,^29^ a validated measure assessing children’s behavioural changes following ambulatory surgery.
- Number of days worked missed by parent(s) or school missed by children, and the reasons including adverse behavioural effects. This provides discrete data and will be used to conduct a social cost analysis

### Independent observations

Independent observations are undertaken before and during induction of anaesthesia by the same observer who is recording the primary outcome, and who is blinded to treatment allocation.

- Children’s compliance during induction: VAS-distress is measured through a 100 mm VAS by the independent observer immediately following observing the induction of anaesthesia (time point 3). This produces continuous data to analyse. No gold-standard tool available for both inhalation and intravenous inductions.
- Children’s distress during induction: VAS-compliance measured through a 100 mm VAS by the independent observer immediately following observing the induction of anaesthesia (time point 3). No gold-standard tool available.

#### Process measures

The case report form (CRF) includes data to be collected for the purpose of comparison between trial arms and description of care processes—these include: premedication (including anxiolysis and/or sedation); type of induction and maintenance of anaesthesia; pharmacological adjuncts (for anaesthesia, analgesia, antiemesis or other symptom control); time to recovery readiness; use of rescue analgesia and antiemetics; unplanned admissions, change in anaesthetic plan and failure to progress with surgery.

#### In-app analytics

For participants in the intervention arm, the app will record the number of times the application is used, including the number of animations triggered and timing of use.

### Data management

Data will be collected from sites using an eCRF (MACRO). Participants are given a unique study subject identifier and subject number.

### Trial end and closedown

A 3-month period for follow-up of patients who have been recruited and randomised will be provided after the date of the last participant recruited. Following that, and data validation, the trial database will be locked for analysis.

### Analysis plan

Data analysis will be overseen by GA as trial lead statistician. Trial arms will be labelled as groups A or B and a postdoctorate research assistant will perform the analysis of the use of the in-app analytics compared with primary outcome measure. Only this team of statisticians will have access to the final trial dataset prior to analysis.

The management of missing data, non-compliers and withdrawals is outlined further in the statistical analysis plan document.

### Primary outcome analysis

We will analyse using an intention-to-treat approach. Our primary analysis will compare mean mYPAS-SF scores at time point 3 (in the anaesthetic room before induction of anaesthesia) between study groups using a mixed-effects regression model with fixed effects for treatment and specialty, and a random effect for centre. All modelling assumptions will be checked, including the assumption of normality for both sets of residuals. If substantial departures from normality occur, then transformations of the outcomes will be considered to achieve normality (of the residuals). Participants who do not contribute any measurements for the primary outcome will be omitted from this analysis.

In addition, the m-YPAS-SF score at other time points will be analysed:

1. The mean score at time point 2 will be compared.
2. The number of participants with a score >30 in either the standard care and intervention arms at time points 2 and 3 will be tabulated and compared.

#### Secondary outcome analysis

In general, for continuous secondary outcomes, we will perform analyses analogous to the analysis of the primary outcome. Otherwise, we will use either the two-sample t-test or Mann-Whitney U test as appropriate. The use of (log) transformations will be considered for time variables since these tend to be skewed. Binary secondary outcomes will be summarised using numbers/percentages and compared, where appropriate, using the χ^2^ test.

#### Subgroup analyses

A subgroup analysis will be performed to investigate the effectiveness of the intervention based on the surgical specialty grouping:

1. Head and neck surgery, dental surgery, maxillofacial surgery.
2. General surgery, orthopaedic surgery, other surgery.
3. Urological surgery, gynaecological surgery.

In addition, a subgroup analysis will be performed to investigate the effectiveness of the intervention according to the age of the patient.

### Other planned analyses

- A priori, we will undertake analyses to explore the impact of issues which arose as a result of the COVID-19 pandemic on trial delivery and/or patient characteristics. This will include the following comparisons of participants who were recruited before and after the COVID-19 pandemic
  - Baseline characteristics including parent and child anxiety levels.
  - Time lapsed between randomisation and primary outcome data collection.
  - Compliance with trial protocols including use of the intervention.
  - Subgroup analyses of outcome data.

### Missing data

The proportion of participants missing outcome data will be summarised by treatment arm. The baseline characteristics of those missing follow-up information on the primary outcome will be compared with those with complete follow-up.

We will investigate the impact of missing values in the primary outcome in two ways:

1. Multiple imputation (using chained equations) may be used to impute missing values of the primary outcome using information collected at other time points. The primary analysis will then be repeated.
2. A mixed model will be fitted that includes outcome data from all time- points.

### Sample size calculation

Local validation of the m-YPAS-SF scoring tool was performed at University College London Hospital. A total of 39 patients, requiring a general anaesthetic for day-case surgery, were observed in the anaesthetic room (time point 3). This showed that 67% of participants displayed significant anxiety on the day of surgery—defined as a m-YPAS-SF score of greater than 30, with a mean m-YPAS-SF score of 37.9 in the anaesthetic room (time point 3), similar to recent published literature^30 31^ and our feasibility trial results. These patients would represent the control arm in the RCT.

The original sample size of 304 patients was calculated to detect a 7.5-point difference in m-YPAS-SF scores between the control and intervention arms at the 5% significance level with 90% power. This assumed a common SD of 26, obtained by combining data assessing the results of mYPAS-SF scores of 3798 children.^26^ Use of this SD over the scores seen in the feasibility trial (15.6 and 7.4 in the standard care and intervention group respectively) was chosen to ensure that the study was not underpowered due to any variability in SD between sites. In addition, the sample size calculation was based on a primary analysis that was adjusted for baseline m-YPAS-SF score and assumed a correlation of 0.7 between the baseline and follow-up values. An attrition rate of 15% was also assumed.

### Interim analysis and revision of sample size estimation and trial protocol

We planned to perform an interim analysis following the recruitment of 100 participants to the trial, to check that the baseline rates and variability in m-YPAS-SF which have guided our sample size calculations hold true in our expanded study population. Due to the pandemic, this was performed early with 119 participants recruited and 67 participants having completed their primary outcome data collection. The primary outcome was analysed with a statistical stopping rule for safety only. This analysis was carried out using a nominal 5% significance level with an O’Brien-Fleming adjustment (within a Lan-de Mets framework). This identified no evidence of harm or need to stop the trial.

However, this analysis revealed a poor correlation between the baseline and preanaesthesia m-YPAS scores. Consequently, the sample size was recalculated without the baseline adjustment, resulting in a revised sample size of 596 patients. The baseline, prerandomisation mY-PAS-SF score was also dropped from data collection.

### Trial start and end dates

The study opened to recruitment in August 2019, and the first patient was recruited and randomised on 19 September 2019. The end date was initially planned to be March 2021; however, the trial was paused between March 2020 and February 2021 because of the COVID-19 pandemic and the cessation of some elective surgical activity and most non-COVID-19-related research activity in the NHS. As described above, the interim analysis which took place in 2020 led to an increase in the planned sample size, which necessitated a further extension to participant recruitment to April 2024; the final participant was recruited on 31 March 2024 and follow-up completed in May 2024.

### Trial management

#### Trial management group

The day-to-day management of the trial is facilitated by a TMG, chaired by the CI which meets a minimum of monthly, and more frequently for cause.

#### Trial steering committee and data monitoring committee

The role of the TSC is to provide independent advice to the TMG and chief investigator, including on approaches to improve the efficiency and feasibility of the trial. The role of the DMC is to provide advice on data and safety aspects of the trial, but where not all members are independent. Meetings of these committees are held approximately once a year, or more frequently if required, to review interim analyses, or as necessary to address any issues. The DMC reviews recruitment and safety data, ensuring appropriate amendments/actions for the trial as necessary.

#### Reporting of adverse events and unintended effects of trial interventions

Expected adverse events related to the use of the VR headset include dizziness, headaches, blurred vision and nausea/vomiting. All other serious adverse events will be reported to the trial management team within five working days of becoming aware of the impact.

#### Patient and public involvement

Patients, carers and the public have been involved throughout the development of the study protocol (including the feasibility study). The PIS, consent form and other patient-facing materials were developed and approved across four National Institute for Health Research (NIHR) Young Persons Advisory Group (YPAG) meetings. There is patient and public involvement representation on the Trial Steering Committee and Trial Management Group, and the TMG Patient and Public Involvement and Engagement representative will contribute to the study report and dissemination of the research findings.

## Ethics and dissemination

The study was approved by the Surrey Borders—Research ethics committee 19/LO/0255, UCL research office as trial sponsor and the Health Research Authority (IRAS 251219) prior to its commencement. The trial is registered with ClinicalTrials.gov NCT03797716 and UCL data protection Z6364106/2018/11/20. All minor and substantial protocol amendments are reported to the sponsor prior to resubmission to the research ethics committee. The study is adopted onto the National Institute of Health Research Portfolio, providing assistance with screening, enrolment, randomisation and data collection (CPMS no: 41142).

The results will be submitted to a peer-reviewed, international journal for publication. Further submissions will be made for secondary outcomes. Dissemination and knowledge mobilisation will be supported by conference attendances, lectures and infographics and engagement with key stakeholders including Royal Colleges, NHS leaders and international colleagues.

## Discussion

Paediatric perioperative anxiety is an important variable that impacts on physiological and psychological outcomes of surgery, as well as having cost implications to hospitals. This study will provide positive or negative high-quality evidence of the effectiveness of a new psychological preparation tool for both parents and children, delivered in the patient’s own home, transforming the way patients are prepared for surgery.

The trial protocol has changed significantly over the course of the study, in part because of the COVID-19 pandemic and consequent changes to paediatric surgical pathways. In particular, the move to remote preoperative assessment in most cases has led to changes in the screening, recruitment and consenting processes, and removal of a baseline, prerandomisation, assessment of anxiety. This measure was initially included for analytic purposes, based on an assumed high correlation with the primary outcome. However, our interim analysis indicated that this correlation was low, and therefore, that the measure was not necessary; as such, the measure was dropped (which assisted in trial delivery, as a face-to-face visit at the time of consent and randomisation was no longer necessary) but the sample size was increased substantially from 304 to 596, including a 15% attrition rate. The post-COVID-19 pandemic changes to healthcare delivery also mean that patients are being offered surgery with much shorter notice than prepandemic. This has led us to reduce the minimum time allowable between randomisation and date of surgery, sequentially over the course of the study, from 4 weeks at the start to 9 days by the end.

The trial has met significant delivery challenges as a result of the COVID-19 pandemic and its consequences; these will be explored further through surveys undertaken with local principal investigators and research staff.

## supplementary material

10.1136/bmjopen-2024-090696 online supplemental file 1

10.1136/bmjopen-2024-090696 online supplemental file 2
