# Supplementary material for: Little Journey: a phase III randomised controlled trial of a psychological preparation and education smartphone application for management of paediatric perioperative anxiety compared with standard care in children undergoing ambulatory surgery – study protocol
Source: BMJ Open. 2025 Feb 26;15(2):e090696. doi: 10.1136/bmjopen-2024-090696 (PMC11865764; doi:10.1136/bmjopen-2024-090696)

Family/Guardian Information Sheet

We would like to invite you and your child to take part in our research study. Before you decide, we would like you to understand why the research is being done and what it will involve.

One of our team will go through this information with you by telephone or a the pre assessment clinic and answer any questions you may have. Talk to others about the study if you wish.

Part 1 tells you the purpose of the study and what will happen to you and your child if you talk part.

Part 2 gives you more detailed information about the conduct of the study.

Ask us if there is anything that is not clear or if you would like more information. Take time to decide whether or not you want your child to take part.

Part 1 – to give you first thoughts about the project

What is the purpose of the study?

Having an operation is a daunting experience, especially as a child. Anxiety begins well before arriving in hospital and is exacerbated by new, unfamiliar environments and people.

We have developed a new technique to prepare children for an operation using a smartphone app that can be used at home before coming to hospital. Children can use the app with a cardboard virtual reality headset to explore the hospital rooms they'll visit on the day of surgery and learn about what will happen from animated characters of staff.

We would like to study if this new preparation tool reduces children's anxiety levels before an operation and improves their outcomes after surgery compared to the current methods we use.

Why have we been chosen?

Your child has been chosen because he/she has been scheduled for an operation in the near future. We hope to have 596 children in this study.

Do I have to take part?

No, it is completely your choice if you wish for you and your child to join this study. It will have no impact on you or your child's ongoing care or any future care. If you agree to take part, we will then ask you to sign a consent form. If your child is 8-12 years old, is able to understand the research and can write their name, they will be asked to sign an assent form if they are happy to take part.

You will be given a copy of the information sheet and the signed consent/assent forms to keep for your records.

Please note, you can still participate in the research trial if you do not have a smartphone. If you are having a face to face pre assessment clinic and are interested in taking part in the research and have a smartphone, please bring this with you to the appointment.

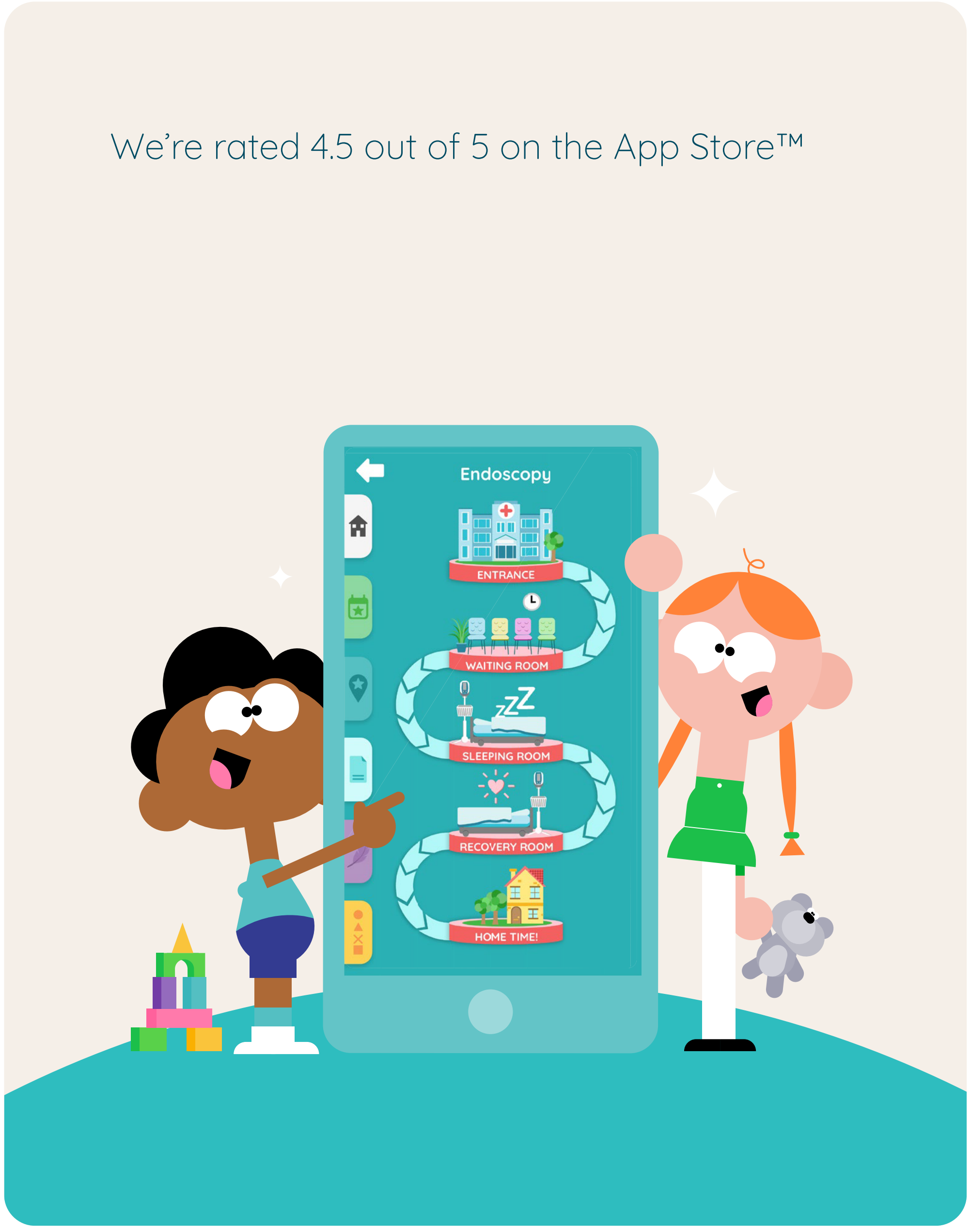

What would taking part involve?

If you are happy for your child to participate in the study, your child will be assigned by a computer to one of two Join on your computer or mobile app groups.

- Group 1: will be given a build-your-own virtual reality headset and code to use with the smartphone app, which you and your child can use in the weeks before their operation.
- Group 2: will be provided with a build-your-own virtual reality headset to use with a variety of free virtual reality apps.

By using a computer to assign your child to a group, which is otherwise known as randomisation, it means there is the same chance that they could be placed in each group. This helps to ensure the process of dividing children into the two groups is fair.

Children in both groups will receive exactly the same care on the day of the operation. On the morning of their surgery, the anaesthetist and surgeon looking after them will visit you both on the ward and discuss with you and your child what will happen.

Your child will be given a hand-held tablet, like an I-pad, to play with in the anaesthetic room to help with the 'going to sleep' process. We encourage you to be present during this. Following their operation, your child will be taken to the recovery room until they are comfortable and awake enough to return to the ward.

As part of the research, we will observe your child's behaviours throughout the day of the operation, recording their anxiety levels on the ward before their operation and in the anaesthetic room. We will also ask you to complete questionnaires assessing your anxiety level following observation of the 'going to sleep' or induction of anaesthesia process. At the end of the day, before you go home, you will be given a patient satisfaction survey to complete.

Finally, we will telephone and/or email to complete a final questionnaire assessment at two- and four-weeks after you go home, which will conclude you and your child's involvement in the research study. Your child can keep the virtual reality headset following the trial to use as they wish with the freely available virtual reality apps available on the app stores.

How do I use the virtual reality preparatory app?

Families allocated to Group 1 will be asked to download the free smartphone app. We will provide you with a password to access the app and encourage you to download it at home. The app has been designed to be easy to use with step-by-step information as you progress through the loading process.

In the weeks leading up to your child's operation, we ask that you allow them to use the app as many times as they want. You can use the app as well, as there is information specifically to help you prepare. The app is designed to be used with a virtual reality cardboard headset which we will send to you by post.

How long will the study last?

If you are happy to take part, your involvement in the study will begin from when you have a confirmed date for the operation and continue until four-weeks after the operation.

Future research

Your information could be used for research in any aspect of health or care, and could be combined with information about you from other sources held by researchers, the NHS or government.

Where this information could identify you, the information will be held securely with strict arrangements about who can access the information. The information will only be used for the purpose of health and care research, or to contact you about future opportunities to participate in research. It will not be used to make decisions about future services available to you, such as insurance.

Where there is a risk that you can be identified your data will only be used in research that has been independently reviewed by an ethics committee.

What other choices do I have if I do not take part in the study?

You are free to choose for you and your child not to participate in the study. If you decide not to take part in this study, there will be no penalty to you or your child and your child will continue to have their operation as planned with the standard care for your hospital.

Little Journey

What if I no longer want, or am able, to carry on with the study?

You are free to stop participating in the study at any point without providing a reason. There is no penalty to you or your child for stopping to participate and the standard of care you or your child receive during this hospital visit, or any subsequent visits, will not be affected in any way. In line with Data Protection Act (2018) and GDPR, any data collected prior to you withdrawing from the study will be kept, but no additional data will be collected once you withdraw. The same applies if you are no longer able to provide consent for you and your child's participation in the trial at any time during the study period.

What are the costs of taking part in the study? Will I be paid for taking part in this study?

There are no costs to you for taking part in this study. You and your child will not be paid for taking part in this study.

What are the possible benefits of taking part?

We cannot promise the study will help you or your child, but the information we get from this study will help improve the care we provide to all children before an operation in the future. Any publications in medical journals as a result of this study will be made available on the SITU website: https://www.ucl.ac.uk/surgery/research/surgical-interventional-trials-unit-situ

What are the risks of taking part?

It is possible that use of the app could worsen your child's anxiety about the operation. However, we believe this is very unlikely as the app has been developed based on the results of similar studies assessing the best ways to provide information to children before an operation and have received great feedback from children and their parents who have used the app.

Potential side effects from using a virtual reality cardboard headset include dizziness, headaches, blurred vision and nausea/vomiting. Based on our pilot study data we expect these to occur in less than 5% of cases and resolve immediately on stopping using the headset.

Will my taking part be kept confidential?

Yes. We will follow ethical and legal practice and all information about you, including any questionnaires you may complete, will be handled in confidence. No personal data will be published at any time and your confidentiality is of the utmost importance to us. Data will be held in secure databases to which only authorised people will have access.

Part 2 – more detail – information you need to know if you still want to take part.

Who has reviewed this study?

All research in the NHS is looked at by an independent group of people, called a research ethics committee to protect your safety, rights, well-being and dignity. This study has been reviewed by the London Surrey Borders Research Ethics Committee. It has also been reviewed and approved by a national research governing body called the Health Regulation Authority.

Who has designed and is organising this study?

The study has been designed by a team of researchers at University College London in conjunction with hospital staff including anaesthetists, nurses and patients. The study is being organised by the UCL SITU (Surgical and Interventional Trials Unit) on behalf of the Chief Investigator for this study, Professor Ramani Moonesinghe and sponsored by University College London. The study has been funded by the National Institute for Health Research (NIHR) Research for Patient Benefit (RfPB) scheme and will contribute towards the PhD of one of the research team.

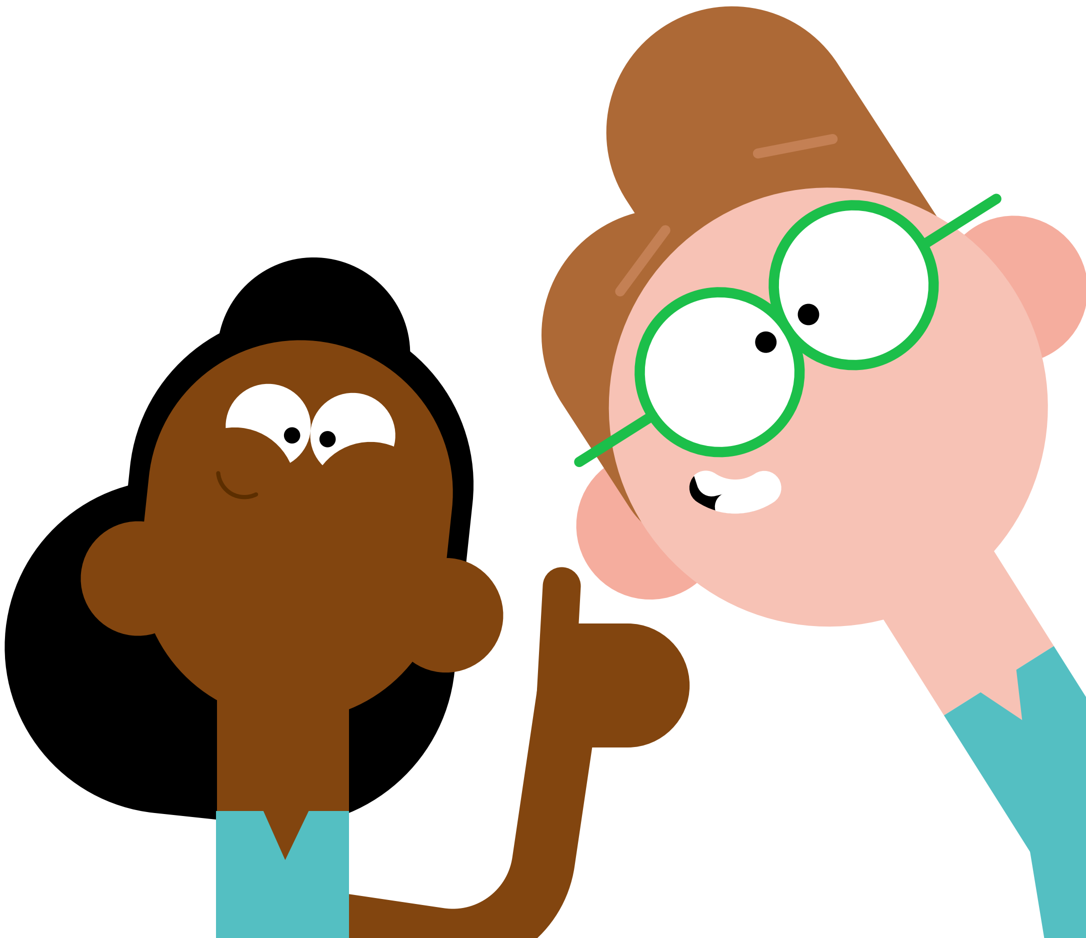

What information will be held about you and what will we do with it?

University College London (UCL) is the sponsor for this study based in the United Kingdom. The sponsor will use information from guardians, children and the child's medical records in order to undertake this study and will act as the data controller for this study.

This means that the sponsor is responsible for looking after your child's/ward's information and using it properly. The sponsor, UCL will keep identifiable information generated by the research for 20 years after the study has finished. This information will be held by [NHS Trust site]

Your rights to access, change or move your/the child's information are limited, as we need to manage this information in specific ways in order for the research to be reliable and accurate. If you/they withdraw from the study, we will keep the information about you/them that we have already obtained. To safeguard your/their rights, we will use the minimum personally-identifiable information possible.

[Your local NHS name here] here will collect information from you and your medical records for this research study in accordance with our instructions.

[Your local NHS name here] will keep your name, NHS or hospital number and contact details confidential and will not pass this information to UCL.

[Your local NHS name here] will use this information as needed, to contact you about the research study, and make sure that relevant information about the study is recorded for your care, and to oversee the quality of the study. Certain individuals from UCL and regulatory organisations may look at your medical and research records to check the accuracy of the research study. The trial team at UCL will only receive information without any identifying information. The people who analyse the information will not be able to identify you and will not be able to find out your name, NHS or hospital number or contact details.

[Your local NHS name here] will keep identifiable information about you from this study for up to 20 years after the study has finished. In addition, the trial co-ordinators, the Surgical and Interventions Trial Unit (SITU), will retain your contact information for a period of one year after the study has finished and may invite you to participate in a one year follow up study about your child's physical and mental recovery after surgery.

If you wish to raise a complaint on how we have handled your personal data, you can contact our Data Protection Officer who will investigate the matter. If you are not satisfied with our response or believe we are processing your personal data in a way that is not lawful you can complain to the Information Commissioner's Office (ICO).

The UCL Data Protection Officer for this trial is Lee Shailer who can be contacted on data-protection@ucl.ac.uk

What if there is a problem?

If you have a concern about any aspect of this study, you should ask to speak to the researchers who will do their best to answer your questions, contact details are at the end of the document. If you remain unhappy and wish to complain formally, you can do this via the hospital's Patient Advisory Liaison Service (PALS).

http://www.nhs.uk/chq/pages/1082.aspx?CategoryID=68

Every care will be taken in the course of this study. However, in the unlikely event that you are injured by taking part, compensation may be available. If you suspect that the injury is the result of the Sponsor's (University College London) or the hospital's negligence then you may be able to claim compensation. After discussing with your research practitioner, please make the claim in writing to Professor Ramani Moonesinghe who is the Chief Investigator for the research and is based at UCLH Department of Anaesthetics (3rd Floor, Maples Link Corridor, University College London Hospital, 235 Euston Road, NW1 2BU). The Chief Investigator will then pass the claim to the Sponsor's Insurers, via the Sponsor's office. You may have to bear the costs of the legal action initially, and you should consult a lawyer about this.

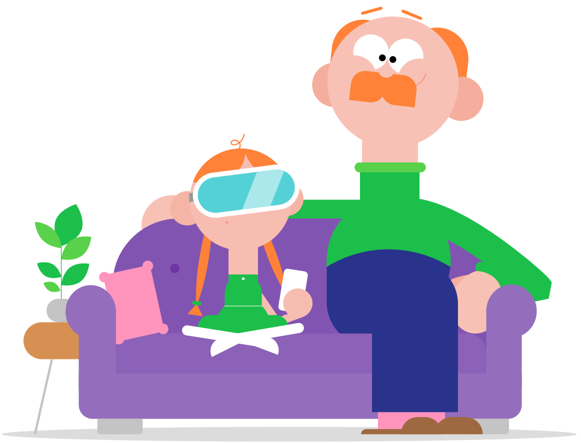

Little Journey

Contact information

You are encouraged to ask any questions you wish, before, during or after your treatment. If you have any questions about the study, please speak to your study nurse, doctor or practitioner, who will be able to provide you with up to date information about the procedure(s) involved. If you require any further information or have any concerns while taking part in the study please contact one of the following people:

Local Lead (Principal) Investigator

Name:

Tel. Number:

Research Practitioner

Name:

Tel. Number:

If you decide you would like to take part then please read and sign the consent form. You will be given a copy of this information sheet and the consent form to keep. A copy of the consent form will be filed in your patient notes and another filed with the study records.

You can have more time to think this over if you are at all unsure.

Thank you for taking the time to read this information sheet and to consider this study.

Giving your consent is voluntary and more information is available if you are unsure.

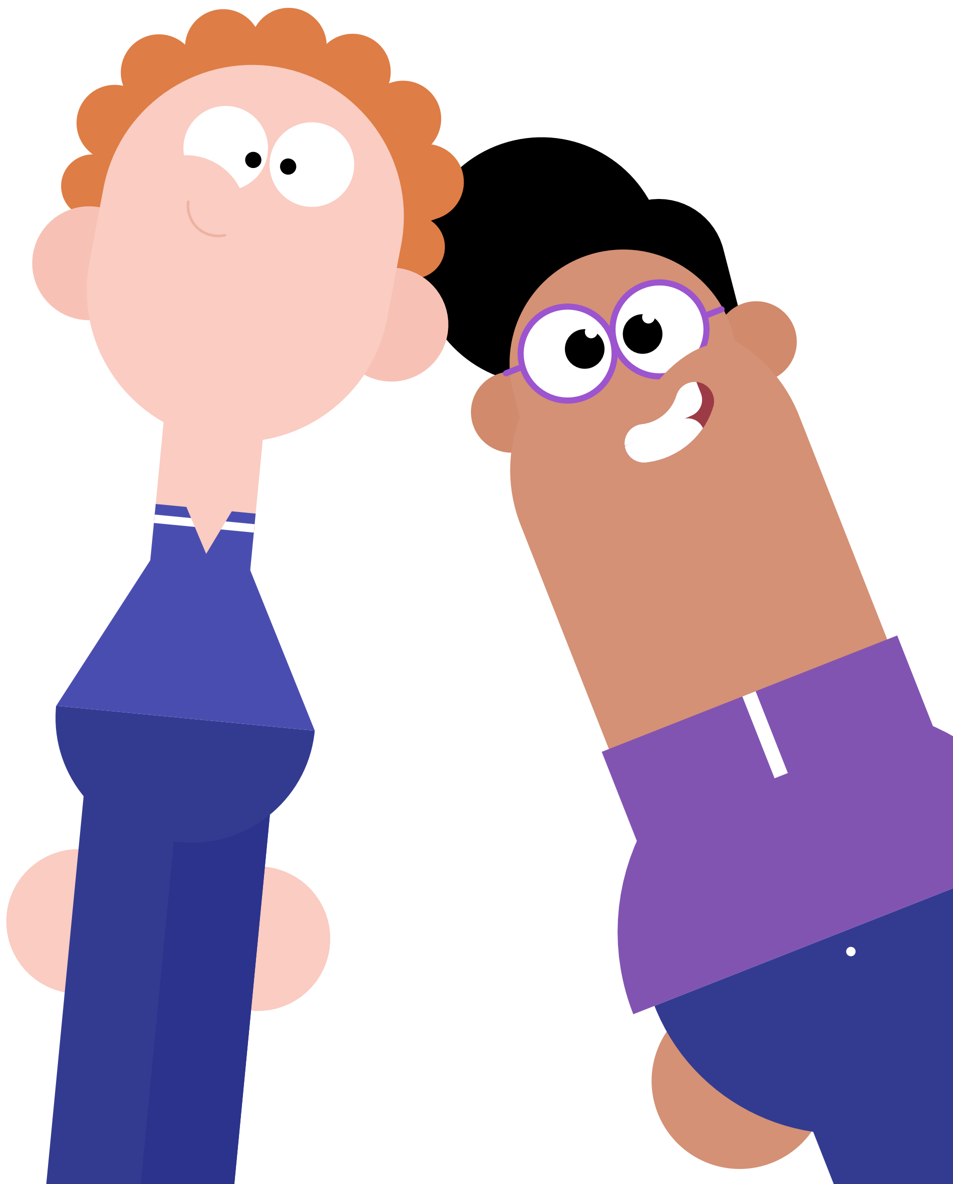

Supplement: online supplemental file 1 [file bmjopen-15-2-s001.pdf]
