# Supplementary material for: Little Journey: a phase III randomised controlled trial of a psychological preparation and education smartphone application for management of paediatric perioperative anxiety compared with standard care in children undergoing ambulatory surgery – study protocol
Source: BMJ Open. 2025 Feb 26;15(2):e090696. doi: 10.1136/bmjopen-2024-090696 (PMC11865764; doi:10.1136/bmjopen-2024-090696)
Supplement: online supplemental file 2 [file bmjopen-15-2-s002.docx]

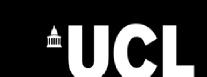


**[Print on Hospital Headed paper)**

**PARENT/LEGAL GUARDIAN CONSENT FORM**

| 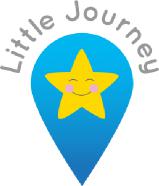 | **Title**: A Multi-site randomised controlled trial assessing the effectiveness of the Little Journey app at reducing peri-operative anxiety compared to standard care  **R&D / Sponsor Reference Number**: 18/0197 **Protocol version number and date***: __________* |
| --- | --- |

**STAGE ONE: TO BE COMPLETED AT TIME OF TELEPHONE CONSENT**

| **Subject Number:** |  |
| --- | --- |
| **Name of Parent/Carer** |  |

**Person Obtaining Consent**

I have read this form to the subject. An explanation of the research was given and questions from the subject were solicited and answered to the subject’s satisfaction. In my judgment, the subject has demonstrated comprehension of the information. The subject has provided oral consent to participate in this study.

| **Name of Researcher obtaining remote**  **consent:** |  | | |
| --- | --- | --- | --- |
| **Date:** |  | **Time:** |  |

When completed: 1 for participant; 1 for researcher site file; 1 to be kept in medical notes.

Little Journey Trial: IRAS No. 251219 Informed Consent Form Version 1.5 06Feb2024

Page **1** of **2**


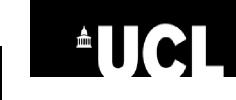


**PARENT/LEGAL GUARDIAN CONSENT FORM**

| 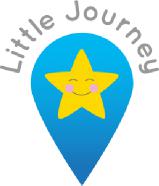 | **Title**: A Multi-site randomised controlled trial assessing the effectiveness of the Little Journey app at reducing peri-operative anxiety compared to standard care  **R&D / Sponsor Reference Number**: 18/0197 **Protocol version number and date***: __________* |
| --- | --- |

**STAGE TWO: TO BE COMPLETED ON THE DAY OF SURGERY FOLLOWING ARRIVAL AT HOSPITAL**

Please initial all boxes

1. I confirm that I have read the information sheet dated __________ (version ____) for the above study. I have had the opportunity to consider the information, ask questions and have had these answered satisfactorily.
2. I understand that our participation is voluntary and that I am free to withdraw at any time without giving any reason, without my medical care or legal rights being affected.
3. I understand that relevant sections of my child’s medical notes and data collected during the study, may be looked at by individuals from regulatory authorities or from the NHS Trust, where it is relevant to my taking part in this research. I give permission for these individuals to have access to the records.
4. I understand that the information collected about my child will be used to support other research in the future and may be shared anonymously with other researchers.
5. I confirm that the Surgical & Interventional Trials Unit can hold my details and contact me after one-year for an update on my child’s recovery after surgery.
6. I agree for my child and I to take part in the above study.

Name of Participant Date Signature

Name of Person Date Signature

taking consent

IRAS Number: 251219 Informed Consent Form Version 1.5 06Feb2024

Page **2** of **2**

When completed: 1 for participant; 1 for researcher site file; 1 to be kept in medical notes.
