# Supplementary material for: Little Journey: a phase III randomised controlled trial of a psychological preparation and education smartphone application for management of paediatric perioperative anxiety compared with standard care in children undergoing ambulatory surgery – study protocol
Source: BMJ Open. 2025 Feb 26;15(2):e090696. doi: 10.1136/bmjopen-2024-090696 (PMC11865764; doi:10.1136/bmjopen-2024-090696)
Supplement: Supplementary file 3 [file bmjopen-2024-090696.reviewer_comments.pdf]

PEER REVIEW HISTORY

BMJ Open publishes all reviews undertaken for accepted manuscripts. Reviewers are asked to complete a checklist review form and are provided with free text boxes to elaborate on their assessment. These free text comments are reproduced below.

ARTICLE DETAILS

Title (Provisional)

The Little Journey: Study protocol for a Phase III randomised controlled trial of a psychological preparation and education smartphone application for management of paediatric peri-operative anxiety compared to standard care in children undergoing ambulatory surgery.

Authors

Evans, Christopher; Bercades, Georgia; Ambler, Gareth; Wilson, Matthew; Brew-Graves, Chris; Baldini, Cinzia; Begum-Ali, Nazma; Williams, Norman R; Emberton, Mark; Fenton, Matthew; Fancourt, Daisy; Samani, Mohini; Mythen, Monty; Moonesinghe, S. Ramani

---

This article was not externally reviewed at BMJ Open. Protocols that have been independently assessed prior to submission to BMJ Open are usually fast-tracked to publication on the grounds that further substantial changes will not be possible. This independent assessment will usually be external, independent review for both a substantial grant award from a non-commercial or government funder and ethics approval.
